# Perceptions of adolescents regarding sexual and reproductive health role models

**DOI:** 10.4102/safp.v68i1.6222

**Published:** 2026-03-10

**Authors:** Tshiamo N. Ramalepa, Thinavuyo R. Netangaheni, Siyanda A. Ngema

**Affiliations:** 1 Department of Nursing Science, School of Healthcare Sciences, Sefako Makgatho Health Sciences University, Tshwane, South Africa; 2 Department of Health Studies, College of Human Sciences, University of South Africa, Tshwane, South Africa; 3 Adelaide Tambo School of Nursing Sciences, Faculty of Science, Tshwane University of Technology, Tshwane, South Africa

**Keywords:** adolescents, perceptions, sexual and reproductive health, role models, role modelling

## Abstract

**Background:**

The phase of transition from being a child to an adolescent is marked by sexual and reproductive health changes that affect the physical, emotional and psychosocial well-being of adolescents. This often influences their thinking, decision-making, lifestyle and choices. Therefore, the presence of role models can enhance sexual and reproductive health outcomes by creating awareness and providing relevant information. This study explored the perceptions of adolescents regarding sexual and reproductive health role models in the Bojanala district, North West Province, South Africa.

**Methods:**

This qualitative, exploratory and descriptive study was conducted using semi-structured interviews. The sample population was 13 adolescents who were sampled purposively. Data were analysed using content analysis.

**Results:**

The themes that emerged in the study are perceived role models, sexual and reproductive health support, and role models in sports and the media. Adolescents identified parents, relatives, peers, media personalities and community members as role models who influence their sexual and reproductive health. Role models in the media and in sports were viewed as people who encouraged focus, discipline and healthy living while keeping adolescents away from risky behaviours.

**Conclusion:**

Adolescents view sexual and reproductive health role models as important mentors who impact their choices and behaviours as they grow into adulthood. It is essential to improve the sexual and reproductive health outcomes of adolescents through incorporating role modelling in families, schools, the media and communities at large.

**Contribution:**

This study contributes to the paucity of knowledge and literature about adolescents’ sexual and reproductive health role modelling.

## Introduction

An adolescent is any person between the ages of 10 and 19 years, during a period of growth and development, who is transitioning from childhood to adulthood.^1,2^ The phase of transition is marked by sexual and reproductive health (SRH) changes that are characterised by physical, emotional and psychosocial changes, which may influence their thinking, decision-making, lifestyle and choices.^2^ On the other hand, role models are individuals who exhibit behaviours and life choices that are appealing to adolescents, encouraging them to emulate these behaviours to achieve their personal goals and desires.^3^ In SRH, role models are important during growth and development because they influence others psychologically and assist them in making important life decisions.^4^ Svanemyr et al.^5^ suggested that the presence of a role model can enhance SRH outcomes by raising adolescents’ expectations for completing their education and expanding their opportunities for earning a living. A role model can be anyone that an adolescent desires to imitate or be guided by; these include parents, peers, teachers, people from the community and people from the media.

Parents and caregivers serve as the main source of guidance and support for adolescents, but promoting their SRH requires active involvement from the wider community. Adams^6^ highlights that parenting becomes particularly demanding during puberty and adolescence, when young people undergo physical, psychological and emotional transitions. Despite these challenges, parents are expected to inform and educate their children, supervise their behaviour and provide support when necessary. By guiding them on sexual risks, shaping attitudes and values, and discouraging risky behaviours, parents play a central role in safeguarding their children.^7^ Nonetheless, adolescents also obtain information from other sources such as the media, which may provide either valuable insights or misleading content.

Mass media is regarded as a source of information for most people, which also applies to SRH information. Health information in general should be accessible to everyone, irrespective of the format it is delivered in. Sserwanja et al.^8^ highlighted that globally, the mass media are recognised as an affordable means of sharing information and have proven helpful in several health initiatives. Some of the health initiatives promoted through mass media include encouraging adolescents to use family planning, raising awareness, sensitising the public and dispelling myths to encourage the desired change in SRH behaviour.^8^ Potential role models from mass media include individual stories covered in the news, celebrities, sportspeople and also individuals that adolescents aspire to, especially regarding their SRH.

Sexual and reproductive health plays a vital role in the overall health and quality of life of individuals, as well as in the social and economic progress of communities and nations. The concept of SRH role models for adolescents is relatively recent and remains underexplored in the South African context. Many adolescents engage in high-risk sexual activities, including unprotected intercourse, having multiple partners and initiating sexual activity at an early age, which increases their susceptibility to unintended pregnancies and sexually transmitted infections (STIs), such as human immunodeficiency virus (HIV).^9^ Statistics from the North West province indicate that between 2020 and 2021, births among girls aged 10–14 years rose by 41%, while those among girls aged 15–19 years increased by 17%.^10^ This upward trend may be linked to adolescents’ limited access to accurate, comprehensive and high-quality SRH information. In response, political leaders in the province have urged measures to protect adolescent girls from harmful sexual influences, abuse and exploitation.^11^ Insufficient awareness of the dangers associated with unsafe sexual practices, combined with barriers to obtaining health information and SRH services, places many young people at risk of negative outcomes. Furthermore, there is little research on adolescents’ perspectives regarding SRH role models. This study, therefore, aimed to explore how adolescents in the Bojanala district of the North West province perceive SRH role models.

## Research methods and design

### Study design

This qualitative, exploratory and descriptive design was used to explore the perceptions of adolescents regarding SRH role models in the Bojanala district, North West province. Exploratory and descriptive research was appropriate for this study because adolescents’ SRH role modelling is a relatively new concept that which little is known.^12^ Furthermore, qualitative research conducted through interviews provided subjective information based on the perspectives and ideas of the adolescents in question.

### Study setting

This study was conducted at a sports complex in a selected municipality in the Bojanala district, North West province. Adolescents were recruited from a municipal sports complex offering facilities for soccer, cricket, rugby, netball, tennis and a gym, which is a popular gathering place for youth. Bojanala district is in the North West province. The district is divided into five local municipalities and has an estimated population of 1 848 133, with 36% of the population being children under the age of 18 years.^13,14^ This district has higher fertility compared to the national average in South Africa.^14^ Many people move to this area for work in the mining industry. Moreover, the population of the selected municipality was estimated to be 536 111 in 2016, and its economy was dominated by mining and agricultural practices.^15,16^ The healthcare crisis in the district is worsened by the triple epidemic of diseases, including HIV/acquired immunodeficiency syndrome, tuberculosis and chronic lifestyle diseases.^14^

### Population and sampling

The population for this study was adolescents. The target population were male and female adolescents in a municipality in the Bojanala district. The accessible population were adolescents coming for sports activities at a local sports complex. Those included in the study were adolescents above the age of 18 years. Non-probability purposive sampling was used to select the participants whom the researchers were interested in because they were deemed knowledgeable about SRH role models.^12^ Participants had prior exposure from school-based programmes such as the Life Orientation lessons or health education sessions covering topics such as contraception, puberty, STI prevention and healthy relationships. They were also involved in youth development activities at the sports complex, which routinely discussed role models and SRH. The researchers were interested in the knowledge, views and preconceptions about SRH role models. Only participants who agreed and consented to participate were included in the study. Participants were approached by the researcher while they were walking around the sports complex and were invited to take part in the study. A few individuals declined participation, indicating that they felt uncomfortable taking part in an interview. The sample size was 13 participants, which was determined by data saturation. Saturation occurred when the researchers conducted the 10th interview and realised that there was no additional information emerging. Then the researchers conducted three more interviews to confirm data saturation.

### Data collection methods

Data were collected using individual semi-structured interviews. Qualitative semi-structured interviews allowed the researcher to obtain responses from adolescents in a face-to-face setting and pose open-ended and closed-ended questions to them while probing to gather more information about SRH role models.^12^ A semi-structured interview guide was used as the data collection tool. The interview guide included two sections, namely, Section A for the demographic data and Section B containing closed-ended and open-ended questions to get in-depth information on the participants’ perspectives regarding SRH role models. Section A addressed participants’ demographics, such as gender and age, and Section B focused on interview questions about the perception of adolescents regarding SRH role models. The main question posed to the adolescents was as follows: *What are your perceptions regarding sexual and reproductive health role models?*

The data were collected between November 2023 and February 2024. Before data collection, the proposal was approved by the College Research Ethics Committee (CREC), College of Human Sciences, University of South Africa. Furthermore, the researcher obtained permission from the municipality. To access the sports complex, the researchers approached the sports office at the municipality and were referred to by the municipal manager, who provided permission to conduct the study. Appointments were made with adolescents who were interested in participating in the study. Adolescents were given the information leaflet to familiarise themselves with the study processes and what was required of them. Only participants who agreed and signed the consent form were included in the study. The interviews were conducted in the office at the gym building, and lasted for 30 min to 40 min. Each participant was provided with a consent form to sign. Verbal consent was also obtained for recording the interviews. The interviews were conducted in English and transcribed verbatim so that data analysis could be conducted. The researcher had prior experience as a soccer coach; however, he did not coach the participants involved in this study and therefore did not hold a direct supervisory or authoritative role over them. While the researcher was significantly older than the adolescents, they shared the same cultural background, which helped establish rapport and ease communication during data collection. English was used as the primary language for the interviews because all participants were able to communicate effectively in English, consistent with their schooling context, where English is commonly used as a medium of instruction. In instances where participants struggled to express themselves fully, Setswana, spoken by both the researcher and participants, was used to ensure clarity, comfort and accurate representation of their views. The researchers performed a pre-test to make sure that the interview guide was suitable for conducting the interviews and that the questions were appropriate to answer the objectives of the study. The three pre-test interviews were subsequently included in the main sample because there were no errors or inconsistencies found by the researcher.

### Data analysis

Data analysis was performed using qualitative content analysis according to Creswell’s six steps of qualitative data analysis.^17^ Content analysis was used to organise and incorporate narrative information from the interviews into themes and sub-themes to provide the perceptions of adolescents regarding SRH role modelling.^18^ During the first step, the researchers prepared and organised the data through transcribing all the interviews verbatim to facilitate analysis. The transcribed interviews were in the form of transcripts. After the transcriptions, the data were cleaned by addressing grammatical errors and language issues. Areas in the transcripts that were not analysed were highlighted; these include short, meaningless phrases and the researchers’ words. The researchers checked and read all the transcripts to get the general tone of the ideas, understanding and the overall sense of the data. The second step was repeated to make sure that the understanding was clear. To improve understanding of the data’s meaning, the researchers made some notes on the side so that the coding process could be facilitated.

Coding was conducted in the third step to start producing meaning; the data were organised into chunks that are related to each other. Similar phrases and words in the transcripts were given a letter combination as codes to ensure that the researchers kept track of similar information across all the transcripts. Different codes started to emerge, and some kept recurring consistently in different transcripts. Grouping and naming of the codes were done, leading to the fifth step of generating a detailed description of the sub-themes. The sub-themes were then grouped to make themes. In the final step, the themes were finalised to ensure meaningfulness and drive a narrative about the perspectives of adolescents regarding SRH role modelling. The researchers then send all the transcripts and audiotapes to an independent coder who conducts another coding process. Then the researcher and the independent coder met to discuss their findings and make comparisons. This consensus meeting led to the conclusion of the themes and sub-themes, supported by verbatim quotes from the participants’ responses.

### Ethical considerations

This study was approved by the College Research Ethics Committee (CREC), College of Human Sciences, University of South Africa. The ethical clearance number is Rec-240816-052. Permission to conduct the study was approved by the office of the municipal manager in the selected municipality. The sports office in the municipality provided access to the sports complex. Informed consent was obtained from the participants after receiving full information about the study. Participants were given the right to withdraw from the study at any time during data collection. The principles of non-maleficence, beneficence and confidentiality were upheld in this study.

## Results

The participants’ demographic information was described by their age and gender. To protect the identities of the adolescents, the researchers used pseudonyms. There were a total of 13 participants in this study, three female and 10 male. Six adolescents were 19 years old, and seven were 18 years old. There were a few female participants because some of them did not want to participate in the study. To personalise the voices of the participants, pseudonyms, gender and age will be used in combination to identify each participant when presenting the findings. For example, *Participant 1: James Male, 18 years*. The three themes that emerged in this study include perceived role models, SRH support and role models in sports and the media. The presented themes below are supported with verbatim quotes from the participants.

### Theme 1: Perceived role models

Adolescents regard SRH role models are people that they look up to and learn from concerning their SRH. They acknowledged that, as young individuals, they behave without considering the results or repercussions of their choices. As adolescents go through puberty and get more knowledge about sexual behaviour and health, SRH role models are crucial in teaching and mentoring them. One participant also mentioned that he would ask his role model questions and get advice when he did not grasp anything. The participants indicated that they look up to their role models because they take care of their sexual behaviour. Some of the participant voices are represented in the following vignettes:

‘These are the people you look up to, the people you look up to when they do things and you want to do what they do, without knowing the consequences of those things.’ (Percy, male, 18 years old)

‘Not that I want to be like him, I just learn a lot from him that might benefit me as well.’ (Thuso, male, 18 years old)

‘Sexual and reproductive health role models are people you look up to, in a type of way that they live their sexual lives and their health regarding their sexual activities, they take care of themselves.’ (John, male, 19 years old)

‘He is someone I can go to when I don’t understand. I ask him and he will guide me the right way, and later I bring him feedback that he helped me a lot.’ (Sanele, male, 18 years old)

Regarding the characteristics of an SRH role model, some individuals held contradicting opinions. One participant thought that if someone commits mistakes in life when they are young, they should not expect to correct adolescents because they made the same mistake. Conversely, another participant valued the opportunity to gain experience from the mistakes made by his role models in their younger years. He was also motivated by the fact that his role model helped him make wiser choices. Some of the participants’ voices are as follows:

‘They are not role models because they waited to reach an age where they are older than said no we should do that while they did it themselves.’ (Percy, male, 19 years old)

‘I would like to talk about it because he has also made mistakes that he is not proud of, so I would say that I would want to be like him in that manner. So, at some point, he has inspired me to at least make better choices that he could not make.’ (Thabiso, male, 19 years old)

### Theme 2: Sexual and reproductive health support

Adolescents mentioned how their various role models support them in their SRH aspects. The participants highlighted mothers, aunts, boyfriends and their peers among the various role models who help. They considered how their role models help them by offering guidance on topics such as abstinence and safe sex practices, as well as by talking to them about their problems. The fact that these people were there for them whenever they needed them was also valued by the participants. One participant was content with the function her boyfriend played in her life and thought of her as a trustworthy individual in times of need. Another participant mentioned that he had a friend who was always accessible to chat about SRH difficulties, making him someone he considered a brother. The participant vignettes that follow:

‘They’ve helped me realise that at my age I should abstain from having sex, as I haven’t had any, so I must say they’ve guided me well because looking at my peers I must say you need to be disciplined and know what you want in life, know what’s right from wrong, choose your influencers wisely and know that engaging in this what will be the outcomes.’ (Sanele, male, 18 years old)

‘I would say my boyfriend [*Laughing*], he is always available, he’s always ready to pitch anywhere anytime whenever I need him, so I would tell him.’ (Kgomotso, female, 19 years old)

‘My uncle has been very helpful to me because he’s the one who advises me to not have multiple partners. I may like them, but not a lot of them.’ (Bofelo, male, 18 years old)

One adolescent described how her mother supported her in her SRH life. She said she discusses everything with her mother. Additionally, anytime she was having difficulties with her SRH, her mother would always reassure her. The participant said the following:

‘My mother because she is the person I can talk to about everything, so when I talk to her like I’m having this problem what’s happening with me she will tell me or she will advise for us to go to the doctor or the clinic, even when I’m experiencing pains before getting my period I sometimes talk to her that I’m having pains and she will reassure me that it’s nothing to worry about it’s common for that to happen it will pass.’ (Kgalalelo, female, 19 years old)

### Theme 3: Role models in sports and the media

According to the adolescents, media and sports figures motivated them to make better choices and be better people. Furthermore, adolescents were thought to be diverted from risky behaviour in the community by participating in sports. A few mentioned that they were able to escape the streets through sports. They emphasised the support and availability of sports coaches as confidants, as well as the importance they played in their lives. They also found that playing soccer prevents adolescents from using drugs and smoking. Adolescents who spent time at the sports complex were able to stay out of dangerous situations while they were out on the streets. The participants said the following:

‘The coaches are not just for football, you can also confide in them and ask for advice, as they’ve experienced life, you can take their opinions on how to go about certain matters.’ (Thabiso, male, 19 years old)

‘The coaches are very much open with me, and they allow me to be free around them and talk about anything that is happening in my life.’ (Kagiso, male, 19 years old)

‘It took me off the street. Many people make mistakes because when they come back from school, they just sit at home. For me, being in soccer, Monday, Tuesday, Thursday and maybe Saturday and Sunday, I am off the street. On Saturday, people are having fun, and I am attending soccer.’ (Percy, male, 19 years old)

The media was also credited with giving adolescents favourable SRH role models; in certain cases, the individuals portrayed as role models help them make better life choices. Adolescents stated that their role models’ behaviour and public personas in some way inspired them to make better SRH choices, even though the identified role models did not disclose their own SRH lives. People in the media have cited discipline, focus, image management, strength and physical fitness as qualities of role models. The participants said the following regarding media role models:

‘The standout is Dr. Bianca and Dr. Kelebogile. They are both female and teach young girls on social media about things to look out for during puberty. Dr Bianca also has an initiative that buys toiletries for young girls.’ (Kgalalelo, female, 18 years old)

‘It boils down to what you consume on the internet, so those people you watch, how do they influence you as they are using that platform to reach out to us? That is how most role models influence us. Those I mentioned before also influenced me through that, I was able to see that as a young man, this is where my focus should be at this stage, which is self-development growth, as there are other stages.’ (Sanele, male, 18 years old)

‘Looking at his age and how he maintains his body, it’s quite interesting as I would also like to keep my body fit for years. I admire the way he built his image, his name, and how he became strong.’ (Paul, male, 19 years old)

## Discussion

The purpose of the study was to explore the perceptions of adolescents regarding SRH role models in the Bojanala district, North West province. The findings reflect that SRH role models are those whom they look up to and can learn from about their SRH. Adolescents in the current study talked about how they behave as young people without considering the consequences or outcomes of their decisions. Since children go through puberty and get more knowledge about sexual behaviour and health, they are influenced by and learn from SRH role models. According to Shansky,^19^ who examined the intricacies of sexual behaviour, reproductive health and cultural impacts on adolescents in several contexts, role modelling plays a multifaceted role in influencing attitudes towards sexuality and reproductive health. The current study revealed that SRH role models helped adolescents by offering guidance on safe sex practices and abstinence, as well as by talking to them about their problems and offering counsel. The fact that these people were there for them whenever they needed them was also valued by the participants.

Role modelling should be a part of SRH for adolescents since they need to be guided in every area of their lives. Sentis et al.^20^ reaffirmed that behavioural and social factors can be explicitly connected to adolescents’ vulnerability to STIs and other risky behaviours. The representations of self, relationships and sexuality that emerge from the interpretation of sexual and relational experiences during this period lay a vital foundation for the development of sexuality throughout adulthood.^21^ According to a qualitative study by Ventura-Miranda,^22^ the primary causes of adolescents’ risky sexual behaviour are their negligence and ignorance of STIs and their negative health effects. Furthermore, adolescent pregnancies and high HIV infection rates in countries like South Africa may also be related to early sexual behaviour, which has been linked to several risk factors, such as having multiple partners and a lack of information and guidance.^23^ By describing the impact that SRH role models play in their lives, the study’s participants affirmed the significance of SRH role modelling.

Ogunbiyi et al.^24^ reflected that the availability of role models within and outside of female adolescents’ homes has a significant impact on their SRH behaviours. The findings of this study demonstrated that when adolescents were unsure about some of the SRH difficulties they were dealing with, they would ask their role models for an explanation. They felt they could look up to these role models since they had more experience and could take care of their SRH. Adolescents in this study mentioned parents, siblings, relatives, soccer coaches, community members and media figures as examples of these role models. A study by Shansky^19^ made the notion that adolescents often seek assistance from a range of sources, including media representations, cultural norms and family dynamics, as they negotiate their own experiences with SRH. Adolescents can imitate unsafe behaviour; thus, the media does not always offer positive role models. Research shows that a small percentage of adolescents admit to participating in behaviours that are commonly the subject of media attention, like being photographed by a partner while having sex.^21^ This means that adolescents may strive to emulate the behaviour of both positive and bad SRH role models they see in the media. However, adolescents in this study only cited media role models who positively influenced their lives.

In the current study, the media was also credited with giving adolescents favourable SRH role models; in certain cases, the individuals who were classified as role models helped the adolescents make better life decisions. Despite their extensive use and influence, experts like Bose et al.^25^ claimed that there is currently inadequate evidence to support the idea that mass media and digital media-based technologies can effectively engage adolescents. This suggests that although SRH information is easily accessible, adolescents might not be using it to inform their choices. The current study recognised a distinct perspective by emphasising that adolescents felt that, in some tiny way, their behaviour and public persona assisted them in making better SRH decisions, even though the selected role models in the media did not disclose specifics about their SRH lives. Strengths, discipline, focus, image control and physical health were among the qualities that teenagers learned from the media.

Adolescents may use sports as a haven to distract themselves from risky behaviour in the neighbourhood. In this study, they reported that participating in sports helped them make better SRH decisions and kept them off the streets. Similarly, adolescent girls in Nairobi’s Dagoretti and Mukuru Informal Settlements have more access to SRHR information, services and empowerment because of Amref Health Africa’s use of sports.^26^ This initiative educated adolescents about SRH through sports. By creating an environment that would improve girls’ safety and empowerment in communities centred around 30 sports clubs, the sports project reached 3000 girls between the ages of 10 and 19, as well as about 9079 community members throughout its 2-year trial phase.^26^ Furthermore, in the context of SHR for adolescents and youth, the importance of creating spaces where young people can access accurate information about their SRHR and youth-friendly services that assist them in making autonomous and responsible decisions regarding their sexuality. In the long run, these programmes can help lower school dropout rates, postpone sexual debut and enhance academic performance, among other aspects of adolescents’ lives, by improving their ability to negotiate and their overall capacity to positively influence access to adolescent and SRH services.^26^ Additionally, the current study found that participation in sports helped decrease drug and smoking usage among adolescents because it kept them out of harmful circumstances while they were out on the streets.

### Limitations

The findings of this study cannot be generalised to adolescents in the district who do not participate in sports clubs. Many adolescents may instead use other social environments as their primary means of socialising and interacting.

## Conclusion

The purpose of this study was achieved by exploring the perspectives of adolescents regarding SRH role modelling at a selected municipality in Bojanala district, North West province. The findings revealed that adolescents recognised a variety of role models who influenced their SRH knowledge, attitudes, and behaviours, such as parents, siblings, peers, community members, sports coaches, and media figures. The ability of role models to offer guidance, advice and support in embracing healthy behaviours like abstinence, safe sex and health-seeking behaviours was highly regarded by adolescents. Influential people in sports and the media were also acknowledged for encouraging attention, discipline and resilience while assisting adolescents in abstaining from dangerous behaviour. This study recommends enhancing positive SRH role modelling at the family, community and institutional levels, considering these findings. While community leaders, educators and coaches can be assisted in incorporating SRH content into their interactions with adolescents, parents and caregivers should be encouraged to speak openly about SRH. Media outlets should also be used to spread SRH-positive ideas and give teenagers trustworthy, relevant role models. This study highlights the crucial role of role models in influencing the SRH of adolescents. To help adolescents make responsible, informed and health-promoting decisions as they transition into adulthood, stakeholders can use the impact of parents, peers, communities, sports and the media.
